# Nephrocast-V: A Deep Learning Model for the Prediction of Vancomycin Trough Concentration Using Electronic Health Record Data

**DOI:** 10.1002/phar.70062

**Published:** 2025-09-30

**Authors:** Ghodsieh Ghanbari, Craig Stevens, Eliah Aronoff-Spencer, Atul Malhotra, Shamim Nemati, Zaid Yousif

**Affiliations:** 1 Department of Biomedical Informatics, University of California San Diego (UCSD) School of Medicine, La Jolla, California, USA; 2 Department of Pharmacy, UC San Diego Health, San Diego, California, USA; 3 Design Lab, University of California San Diego, La Jolla, California, USA; 4 Division of Infectious Diseases and Global Public Health, Department of Medicine, UC San Diego School of Medicine, La Jolla, California, USA; 5 Division of Pulmonary, Critical Care, and Sleep Medicine, UCSD Department of Medicine, La Jolla, California, USA; 6 Skaggs School of Pharmacy and Pharmaceutical Sciences, UCSD, La Jolla, California, USA

**Keywords:** acute kidney injury, critical care, deep learning, machine learning, pharmacokinetics, vancomycin

## Abstract

**Introduction:**

Vancomycin is a critical antibiotic for treating methicillin-resistant *Staphylococcus aureus* and other gram-positive bacterial infections, but achieving and maintaining therapeutic trough concentrations is challenging.

**Objectives:**

We hypothesized that a deep learning model could accurately predict vancomycin trough concentrations 2 days in advance in critically ill patients and provide recommendations for optimal dosing adjustments to achieve target drug concentrations.

**Methods:**

We trained and validated the model using electronic health record (EHR) data from adults admitted to the University of California San Diego Health system intensive care units (ICUs) from January 1, 2016, to June 30, 2024. Features included patient demographics, comorbidities, vital signs, laboratory measurements, medications, and vancomycin dosing information. The model architecture combined Long Short-Term Memory and Multi-Head Attention layers, supplemented with skip connections to incorporate past dosage information at the final layer of the deep learning model. Model performance was evaluated using mean absolute error (MAE) and root mean square error (RMSE) metrics.

**Results:**

A total of 2205 encounters met the eligibility criteria. The median age was 57 years, and the median ICU length of stay was 4.9 days. The model achieved an MAE of 3.15 mg/L and an RMSE of 4.17 mg/L, comparable to that of a critical care pharmacist aided by a Bayesian dosing software. Additionally, deviations from patient-specific model-based dose recommendations were generally associated with nontherapeutic vancomycin levels.

**Conclusion:**

This study demonstrates the potential to leverage deep learning to individualize and support vancomycin therapeutic drug monitoring in critically ill patients.

## 1 | Background

Vancomycin is a glycopeptide antibiotic that has been utilized for over half a century to treat patients infected with gram-positive infections, including those caused by methicillin-resistant *Staphylococcus aureus* (MRSA) [1, 2]. Despite the emergence of alternative antimicrobials, vancomycin continues to be the first line for the treatment of MRSA due to its proven clinical effectiveness and its affordable price [3]. Vancomycin has a narrow therapeutic index, necessitating therapeutic drug monitoring (TDM) [4]. While subtherapeutic concentrations may lead to treatment failure and potentially contribute to bacterial resistance, supratherapeutic levels increase the risk of nephrotoxicity [5].

According to the most recent practice guidelines for vancomycin TDM, in patients with suspected or definitive serious MRSA infections, an individualized target of the area under the curve (AUC)/minimum inhibitory concentration (MIC) ratio of 400–600 is recommended [6]. Although AUC/MIC is considered the pharmacokinetic parameter most closely associated with positive clinical response and reduced nephrotoxicity, its adoption in clinical practice is still incomplete, as this value cannot be directly measured in patients and must be calculated based on serum drug concentration value and exposure over time [7–9]. Additionally, there is insufficient evidence to recommend only *trough* or only AUC TDM of vancomycin in patients with non-invasive MRSA or other infections [6].

Vancomycin dose and frequency are selected based on patient-specific factors to achieve a therapeutic level at steady state. Vancomycin TDM monitoring can be accomplished in two ways. The traditional approach is to measure *peak* concentration 1–2 h after infusion and trough concentration at the end of the dosing interval near steady state. A more recent approach is using Bayesian software to achieve an individualized dose. Both approaches have limitations [6]. Although the first approach requires achieving steady-state conditions and precisely timed blood sample collection, the second approach—using Bayesian dosing software—avoids the need for steady-state trough concentrations but is not without limitations [10]. Bayesian models include a limited number of covariates, such as basic demographics, serum creatinine (SCr) level, vancomycin doses, frequency, and prior concentrations. This results in restricting the models’ ability to comprehensively account for patient-specific factors when predicting vancomycin levels. Existing TDM methods assume a stable kidney function—an assumption often violated in critically ill patients who frequently experience fluctuations in kidney function and acute kidney injury (AKI) [11, 12]. Previous studies reveal that only 34% of critically ill patients achieve therapeutic vancomycin concentrations within the first 48 h of treatment, and fewer than 40% reach the target concentration within 3 days [13, 14]. Clinical pharmacist engagement in vancomycin TDM has been shown to lead to significantly better outcomes, highlighting the importance of incorporating clinical and pharmacokinetic knowledge when deciding a treatment and monitoring plan [15, 16]. This approach, though effective, demands a detailed and time-intensive evaluation of patient-specific factors to determine the optimal dosing regimen.

Unlike traditional statistical methods, machine learning (ML) algorithms can learn patterns from high-dimensional, longitudinal data to construct corresponding models. Using ML for model-informed precision dosing has been a research topic, but it remains in its early stages [17]. Several statistical models have been developed, using ML and deep neural network methods, to predict vancomycin trough concentration in critically ill patients. Although these models have demonstrated continuous improvement in prediction accuracy, limitations exist that hinder their adaptation into clinical practice. These include focusing only on predicting the initial trough concentration, having a short prediction window with limited clinical utility, and a lack of direct comparison with pharmacist aided by Bayesian dosing software [18–20]. To address the limitations of currently existing methods, we hypothesized that sequential ML models, such as recurrent neural networks (RNN) [21], could predict vancomycin trough concentrations 2 days in advance in critically ill adult patients and achieve a performance comparable to that of a critical care pharmacist aided by Bayesian dosing software.

## 2 | Materials and Methods

### 2.1 | Study Cohort

Data were extracted from the electronic health record (EHR) system of the University of California San Diego Health (UCSDH) between January 1, 2016, and June 30, 2024, for model development and internal validation. UCSDH consists of two academic medical centers, including a Level I Trauma Center, which provide critical care across a wide range of specialties, including medical, surgical, cardiovascular, and intensive care units (ICUs). In this study, patients were eligible for inclusion if their age was ≥ 18 years old and they had spent a minimum of 24 h in an ICU. Patient-days were excluded if the patient received continuous vancomycin infusion, kidney replacement therapy (KRT) on the prediction day or the last 7 days from the prediction day, and if the ICU stay extended beyond 14 days. Patient-days were eligible for trough concentration prediction if the patient received at least one intravenous vancomycin dose on the day the prediction was made, and had a corresponding trough level measured 2 days thereafter. The University of California San Diego Institutional Review Board (IRB) approval was obtained with the waiver of informed consent (#800257).

### 2.2 | Outcome Definition

The primary goal is to predict the estimated steady-state vancomycin trough concentration measured 2 days later. Specifically, the model is designed to forecast estimated steady-state vancomycin trough concentration using clinical data available on the prediction day, along with the vancomycin doses administered over the subsequent 2 days leading up to the trough measurement. Vancomycin trough concentrations were extracted from the clinical pharmacy notes using regular expressions. In these semi-structured notes, UCSDH clinical pharmacists document treatment indications, vancomycin trough goals and values, planned vancomycin dosing regimens, and predicted steady-state trough concentration. Pharmacists at UCSDH utilized PrecisePK (San Diego, California, United States), a commercial Bayesian dosing software, to estimate vancomycin trough concentration and tailor patient-specific vancomycin dosing regimens. In this software, pharmacists select the appropriate population pharmacokinetic model and input patient-specific variables, including age, weight, height, SCr, prior vancomycin concentrations, and both past and future vancomycin dosing information. Pharmacists use the TDM parameters predicted by the Bayesian dosing software, along with their clinical judgment, considering the patient’s clinical trajectory and anticipated changes in kidney function, to select the most appropriate vancomycin regimen.

### 2.3 | Clinical Features as Predictors

We considered a comprehensive list of predictors that capture key clinical demographics and comorbidities. We also included vitals, laboratory, and clinical measurements of the patient’s physiological state and illness severity available up until the time of making the prediction. The variables consisted of six demographic features, 62 comorbidities, 53 vital signs and laboratory measurements, 11 Systemic Inflammatory Response Syndrome (SIRS) and Sequential Organ Failure Assessment (SOFA) measurements, and 12 medication features. We also included vancomycin-specific variables, including prior vancomycin concentrations, vancomycin dosage quantities (from the date of prediction until the trough was collected), and time since the last vancomycin dosage. Vital signs and laboratory measurements were compiled at an hourly resolution into non-overlapping bins, with the median value utilized for variables with multiple measurements per hour. Old values were carried forward for up to 24 h if no new measurements were available. All remaining missing variables were imputed using the mean. For each vital sign and laboratory measurement, an additional two features consisting of the slope of change and mean value over the previous 72 h were calculated. For categorical baseline covariates, such as comorbidities, missing values were imputed as not present. We employed a previously developed data pipeline that automates the extraction of data from the Epic Clarity database, processing of clinical features, and imputation of missing values [22].

### 2.4 | Model Development and Validation

**FIGURE 1 |:**
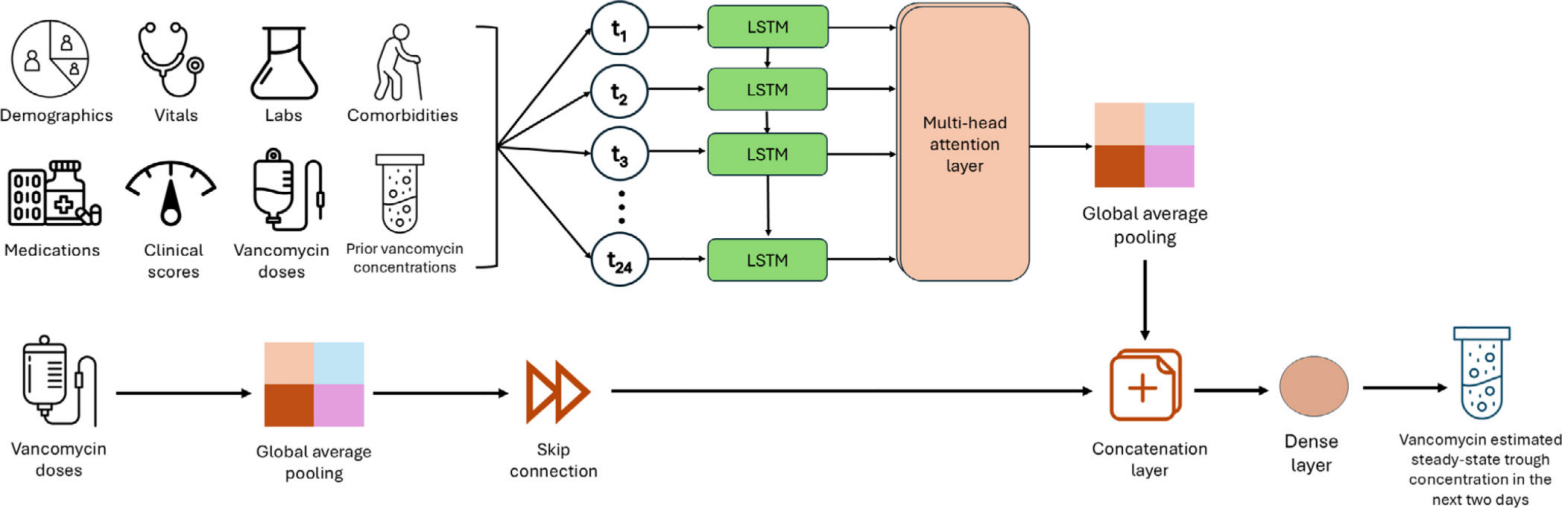
Schematic structure of the Nephrocast-V model. Each *t* represents an hourly time step in a 24-h sequence, where patient data (e.g., vitals, labs, demographics, medications, vancomycin doses, and comorbidities) are fed into the LSTM layer. The model processes these sequential inputs to capture temporal dependencies for predicting vancomycin trough values. LSTM, long short-term memory.

To predict vancomycin trough levels 2 days in advance, we designed Nephrocast-V, a deep learning model with a long short-term memory (LSTM) layer followed by a Multi-Head Attention layer and a final dense layer. The LSTM layer with a hidden state size of 16 captures temporal dependencies within a 24-h sequence of clinical and dosing data, providing hidden states that serve as input to the Multi-Head Attention layer. With two heads and a key dimension of 64, the attention layer operates across the time axis, allowing the model to focus on relevant patterns within each day’s data. Finally, the dense layer with one unit outputs the predicted trough level for each sequence. To enhance the integration of dosage information, the model incorporated skip connections akin to those used in ResNet [23]. These skip connections directly fed past dosage features to the final dense layer, bypassing intermediate layers. This design ensures that critical dosage information remains accessible to the output layer without being diluted through the network’s deeper layers. The dataset was split into 80% for model development (training dataset) and 20% for validation (test dataset). The schematic diagram of Nephrocast-V is shown in Figure 1. Model training was conducted using L2 regularization and the Adam optimizer, with hyperparameters optimized through Bayesian hyperoptimization [24, 25].

### 2.5 | Evaluation of Predicted Trough Concentrations

To evaluate the performance of the model, we compared vancomycin trough concentrations predicted by Nephrocast-V versus the measured steady-state trough concentration estimated 2 days later. Prediction errors were assessed using mean absolute error (MAE), root mean squared error (RMSE), and mean absolute percentage error (MAPE) (Appendix A). Those metrics were benchmarked against predictions made by a clinical pharmacist aided by a Bayesian dosing software. Additionally, we compared the accuracy of our model in predicting whether the vancomycin trough would fall within subtherapeutic, therapeutic, or supratherapeutic ranges based on therapeutic trough goals extracted from the patient’s notes. We compared the concordance of these predictions with those made by a clinical pharmacist aided by a Bayesian dosing software. In cases in which clinical pharmacists decided to dose patients based on AUC rather than trough goals, we utilized documented trough targets from documentation to allow for a consistent comparison.

Bland–Altman plots were used to assess the difference between predicted and measured vancomycin trough concentrations. We performed error analysis of selected cases in which Nephrocast-V exhibited high prediction errors. These cases underwent a comprehensive clinical review by a pharmacist to determine the factors contributing to the prediction error.

Feature importance was determined using gradient-based relevance scores, which measure the contribution of each input feature to the final prediction output. The relevance score was computed by taking the derivative (gradient) of the predicted vancomycin trough level with respect to all input features and multiplying it by the input values [26].

### 2.6 | Model-Based Dosage Recommendations

To evaluate the effectiveness of Nephrocast-V in recommending vancomycin dosing to enhance precision and achieve therapeutic trough levels, we implemented a line search approach to identify the best dose. For predicted trough levels below the therapeutic range, we considered increasing the dosage by 250 mg increments. This process continued until the total increase reached 1500 mg. For predicted trough levels exceeding the therapeutic range, we considered decreasing the dosage by 250 mg decrements and then predicting the trough level using the new dosage. This adjustment continued until either the total reduction exceeded 1500 mg or the dosage reached zero. When the predicted trough levels fall within the therapeutic range, we explored increases and decreases in dosage to fine-tune the recommendation. We limited the change in doses explored to 1500 mg to ensure the clinical relevance of the dose recommendation, as larger dose differences could reflect a significant deviation between the actual and predicted doses, warranting further clinical assessment in those cases.

We employed a reward function that assigns values based on the proximity of predicted trough levels to the therapeutic range to demonstrate that using dose values closer to the model’s recommendations results in trough levels that are more likely to fall within the therapeutic range (Appendix B) [27].

### 2.7 | Statistical Analysis and Software

Patient characteristics were summarized using descriptive statistics such as mean (standard deviation [SD]), median (interquartile range [IQR]), or counts (%), where appropriate. Continuous variables were analyzed using the Wilcoxon rank-sum test, and categorical variables were analyzed using chi-squared or Fisher’s exact test. The difference in MAE, RMSE, and MAPE between Nephrocast-V and pharmacist prediction of vancomycin trough concentrations for the same measurements were compared as paired data and were analyzed using paired samples *t*-test. McNemar’s test was used to compare the prediction of the trough therapeutic category between Nephrocast-V and clinical pharmacist. Significance levels were set at the 5% level. Python 3.10.9 was used for analysis. NumPy 1.23.5 was used for all data preprocessing. The deep learning model was implemented using TensorFlow 2.13.0 [28].

## 3 | Results

### 3.1 | Study Population

A total of 2205 encounters met the eligibility criteria for the study, corresponding to 2492 trough measurements. The cohort demographics are summarized in Table 1. Males represented 63.3% of the training cohort with a median (IQR) age of 57.9 (43.4–67.6) years. In the training cohort, the most common ethnicity was White (52.2%). The median (IQR) baseline SCr was 0.63 (0.48–0.81) mg/dL. About 3.1% of patients had a diagnosis of chronic kidney disease. The rates of AKI stages I, II, and III were 29.0%, 11.9%, and 2.4%, respectively. The median (IQR) days of ICU stays was 4.9 (2.8–7.9). The median (IQR) SOFA II score was 6 (4–9), and the mortality rate during the hospital stay was 8.9%.

### 3.2 | Model Performance

**FIGURE 2 |:**
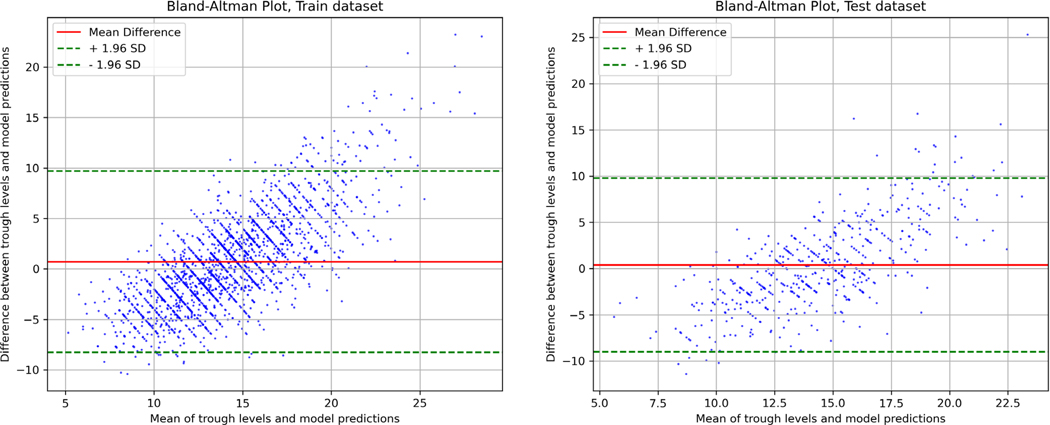
Bland–Altman plots of predicted and measured vancomycin trough concentrations in the train and internal test datasets. SD, standard deviation.

Nephrocast-V achieved an MAE of 3.15 mg/L, RMSE of 4.17 mg/L, and MAPE of 20.45 in the internal test dataset. In comparison, the pharmacist’s predicted MAE, RMSE, and MAPE were 3.06 mg/L, 4.07 mg/L, and 18.60, respectively. The differences in MAE, RMSE, and MAPE between Nephrocast-V and the pharmacist’s prediction were statistically insignificant (Table 2). The distribution in Nephrocast-V prediction error was high for supratherapeutic vancomycin trough concentrations (Figure 2). A similar prediction error pattern was observed in pharmacist dosing aided by Bayesian software (Figure S1).

The therapeutic ranges for vancomycin troughs were usually 10–15 or 15–20 mg/L. In the test dataset, 60.5% of trough measurements were within the therapeutic range, 37.1% were subtherapeutic, and 18.5% were supratherapeutic. The accuracy of the pharmacist’s predicted vancomycin trough concentrations was 56.7% compared to 55.6% of Nephrocast-V (*p* = 0.24, Table 3). Analysis of cases with high prediction error revealed common themes, including AKI onset and resolution, low muscle mass, malnutrition, obesity, and low SCr at baseline (Table 4).

### 3.3 | Feature Importance

**FIGURE 3 |:**
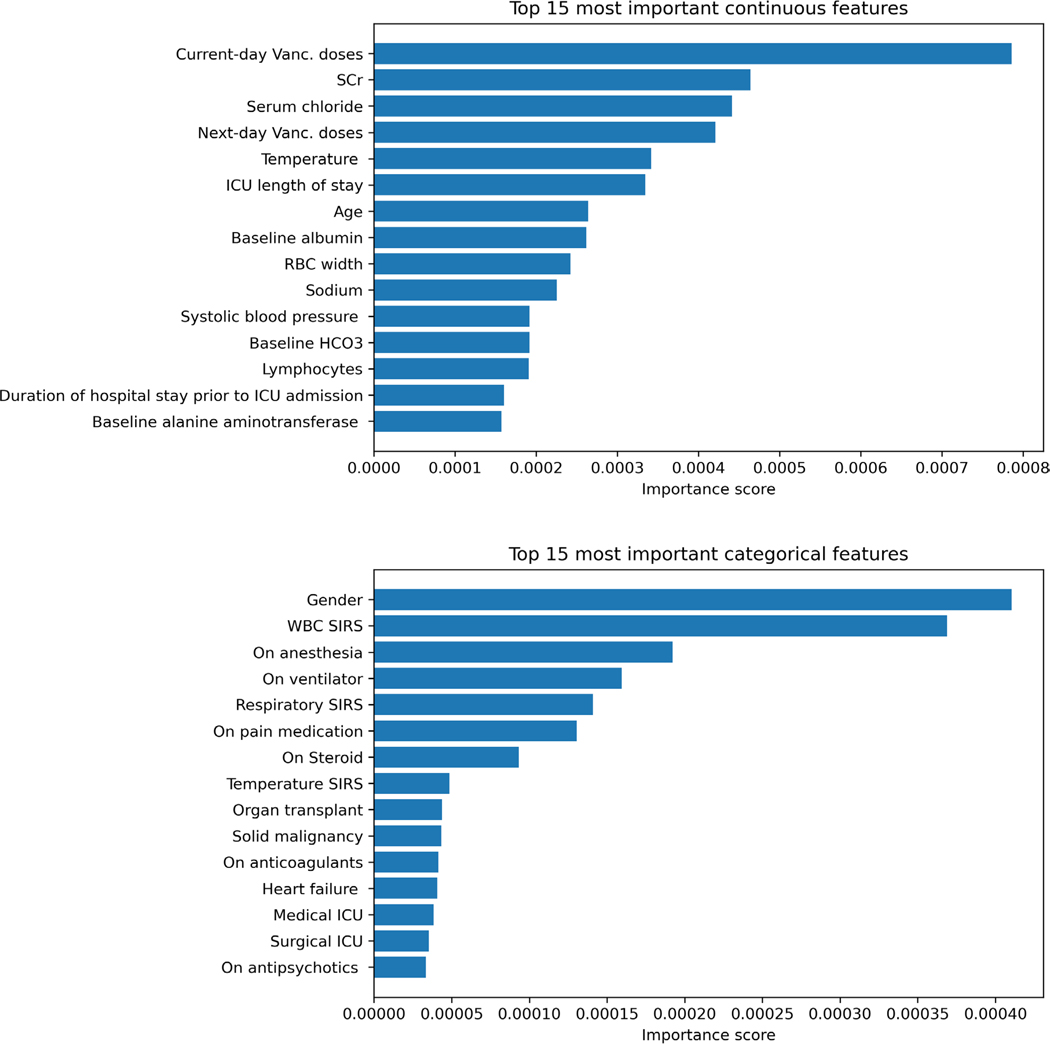
Top 15 features ranked by importance, categorized into continuous and categorical feature groups. HCO3, bicarbonate; ICU, intensive care unit; RBC, red blood cell; SCr, serum creatinine; SIRS, Systemic Inflammatory Response Syndrome; WBC, white blood cell.

A total of 253 features were assessed (Table S1). The top 15 most important continuous and categorical features are shown in Figure 3. The most important continuous feature was the prediction day dose of vancomycin, followed by SCr. In total, two features were related to vancomycin dosage, one related to kidney function, two related to length of hospital stay, and the remainder were related to other laboratory measurements. The most important categorical feature was gender. Three features were components of the SIRS score, and two features were related to the ICU unit specifically. The remaining features were related to comorbidities and medical conditions.

### 3.4 | Dosage Recommendation

**FIGURE 4 |:**
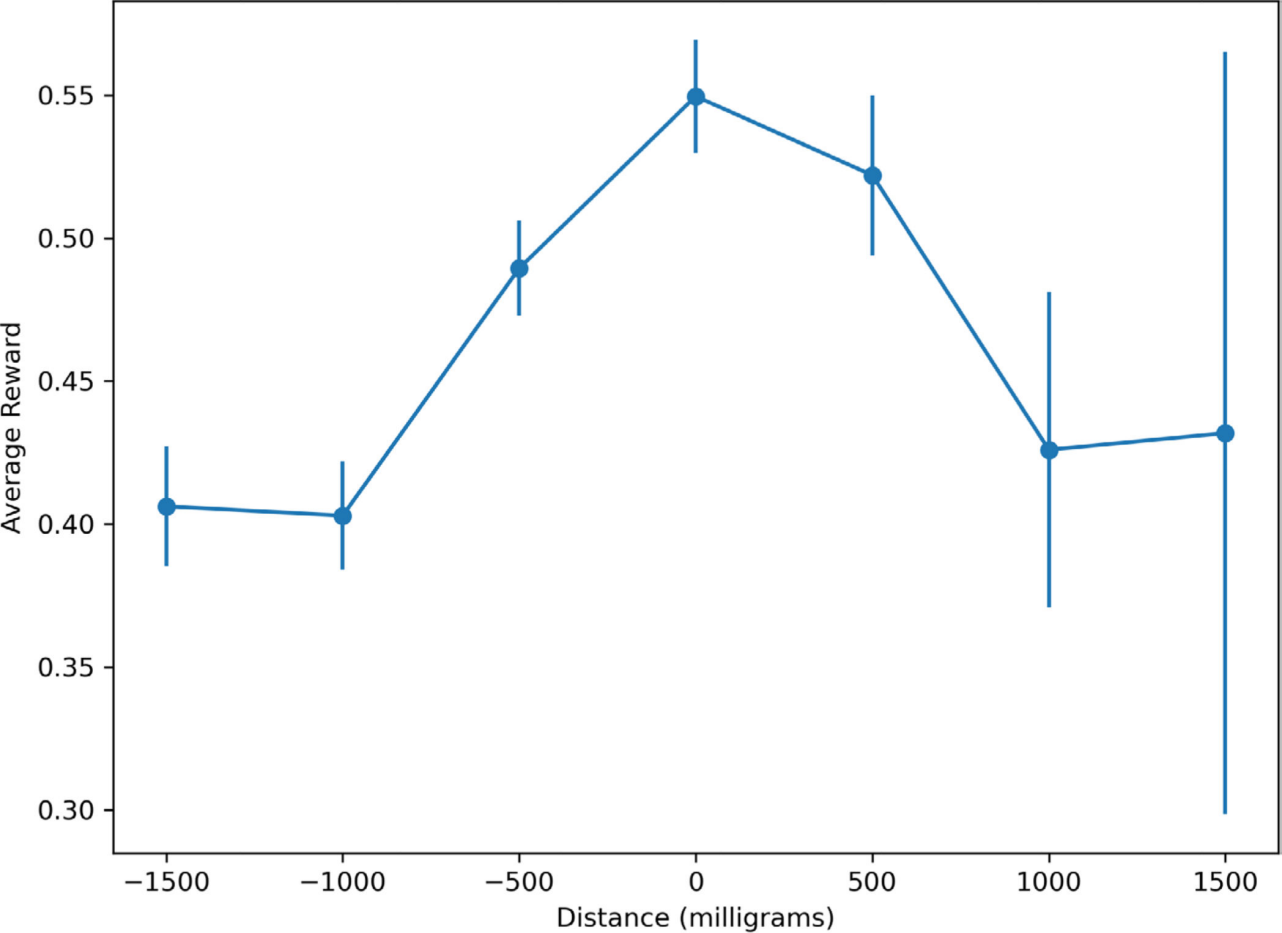
The difference between actual and model-recommended doses (distance) in milligrams and the average reward. A negative distance indicates that the administered dose was lower than the model’s recommendation, while a positive distance indicates that the dose was higher.

The mean difference between the actual and model-recommended doses (Distance) and the corresponding average rewards is shown in Figure 4. The error bars in the plot represent the standard error of the average rewards. The average rewards were highest when the mean difference between the Nephrocast-V recommended dose and the pharmacist-ordered dose was zero, indicating that doses closer to the model’s recommendations resulted in vancomycin trough levels closer to the therapeutic range. Conversely, as the discrepancy between the actual and recommended doses increased, the reward decreased, reflecting that deviations from the model-recommended doses led to trough levels further from the target range. However, the reward function demonstrated a plateau for dose differences below −1000 mg and above 1000 mg.

## 4 | Discussion

We developed an ML model to predict vancomycin trough concentrations measured 2 days later in critically ill adult patients based on a comprehensive data set derived from EHR data, including demographics, comorbidities, vital signs, laboratory measurements, prior vancomycin trough concentrations, and vancomycin dosing. Our model demonstrated comparable performance to that of a clinical pharmacist aided by a Bayesian dosing software, with accuracy exceeding that reported in previous ML studies [13, 14]. Additionally, our model tolerated missing values and demonstrated consistent performance across the range of vancomycin trough concentrations. This work could aid clinical pharmacists by automating the assessment of relevant clinical predictors and providing patient-specific vancomycin dose recommendations.

Although ML has made significant strides, its application to individualized medication dosing remains in the early stages. Our work was centered on creating a model with practical value in clinical settings, while improving on the limitations of existing approaches. In clinical practice, selecting the appropriate vancomycin regimen requires careful consideration of the patient-specific target trough concentration, ensuring that the predicted steady-state trough falls within the desired range. Vancomycin target ranges can often vary based on the infection site and severity. For most patients, a target range of 10–15 mg/L is recommended. Higher target trough levels of 15–20 mg/L are recommended for severe infections [30]. ML models constrained by a fixed therapeutic range, such as 10–20 mg/L, don’t offer patient-specific optimization of dosing targets [19]. Although the prediction of current vancomycin concentration can help monitor patients experiencing acute clinical changes [20], the practical utility of these recommendations is limited since forecasting vancomycin steady-state trough concentration is a prerequisite for selecting the appropriate vancomycin dose and interval. When developing Nephrocast-V, we avoided making any assumptions about vancomycin trough goals, and the assessment of model accuracy was based on patient-specific trough goals. Furthermore, Nephrocast-V predictions were not limited to the first steady-state vancomycin trough concentration or to patients who initiated their vancomycin treatment in the ICU. Our prediction window of 2 days aligns with general clinical practice, allowing sufficient time for the patient to reach, at minimum, a pseudo steady-state before measuring the trough concentration.

Previous research has highlighted several domains in which ML could impact clinical pharmacy practice [31]. Despite advances in clinical informatics, TDM remains a labor-intensive process, requiring manual data entry, interpretation of laboratory measurements, and assessment of clinical trajectory to guide dosing decisions. A time and motion study estimated that clinical pharmacists spend approximately 20% of their clinical time gathering and evaluating patient data [32]. Another study estimated that manual data entry for vancomycin TDM can take up to 15 min per patient [33]. Automating these steps with our model has the potential to significantly reduce the effort required by clinical pharmacists to dose vancomycin while maintaining the quality of the current process. By leveraging Fast Healthcare Interoperability Resources (FHIR) and Health Level Seven (HL7) standards, data has been successfully extracted from the EHR in real time, allowing for the implementation of ML algorithms in clinical settings [22].

Our evaluation of Nephrocast-V high-importance features aligns with previous models. Unsurprisingly, variables related to kidney function, such as SCr, creatinine clearance, or estimated glomerular filtration rate, scored highly across all the models [19, 20, 34, 35]. About 80% to 90% of a vancomycin dose is excreted in urine as the unchanged drug, and its clearance has been shown to be correlated with creatinine clearance [36]. Variables related to vancomycin dosing ranked highly in our model, demonstrating the importance of dose selection for our model predictions and its dose recommendation feature. Predictors of critical illness, such as SIRS score, ventilation, and ICU length of stay, ranked highly among other variables. This highlights the multifactorial nature of vancomycin pharmacokinetics and the importance of incorporating diverse clinical data for accurate predictions [37].

The prediction error in Nephrocast-V was higher for supratherapeutic vancomycin trough concentrations, which can be attributed to two factors. First, vancomycin trough concentration values don’t have a maximum value. As a result, high supratherapeutic concentrations are more challenging to predict compared to therapeutic and subtherapeutic concentrations. Second, in our dataset, supratherapeutic vancomycin concentrations were less common compared to therapeutic and subtherapeutic levels, leading the model to be trained on fewer high-concentration examples. The semi-connected diagonal lines observed in the Bland–Altman plots can be attributed to the distribution of vancomycin trough concentrations in our dataset, where most values were rounded to whole numbers or one decimal place when recorded in the clinical notes.

Error analysis reveals common themes related to kidney function assessment. Creatinine is the end product of creatine and creatine phosphate metabolism from muscle and protein metabolism [38]. It has significant limitations as a biomarker to assess kidney function and evaluate AKI onset and resolution. Low SCr levels are associated with conditions, including low muscle mass, malnutrition, amputation, and muscle wasting, leading to the overestimation of kidney function [39–41]. These clinical conditions were prevalent in our review of cases with high prediction errors. Rounding SCr to 1 for values below 1 mg/dL is a common practice in some institutions; however, this approach has been shown to potentially increase the risk of underestimation of renal function in certain scenarios, potentially impacting the accuracy of medication dosing [42, 43]. In AKI, SCr changes lag behind the actual timing of the kidney insult and resolution by 48–72 h [44]. This could result in misestimating renal clearance and vancomycin steady-state trough concentration. Several biomarkers have been evaluated as potential alternatives or adjuncts to SCr. Cystatin C (CysC) is a low molecular weight protein that is produced constantly by all nucleated cells. Unlike SCr, CysC is less affected by sex, muscle mass, and nutritional status. Despite its advantages, the utilization of CysC continues to be low compared with SCr in critically ill patients [45]. Recent guidelines have recommended estimating kidney function using CysC in subpopulations in which SCr is a poor biomarker [46].

We note limitations that will require refinement and future investigation. First, the data were derived from a single health system, potentially limiting generalizability to other populations or health care settings. Additionally, although the model performed well in internal validation, external validation across diverse clinical environments is needed to confirm its utility [47]. Second, the exclusion of patient-days with KRT on the predication day or the last 7 days, and after 14 days from the ICU admission date might have contributed to the risk of selection bias and further limited the generalizability of this work. The plateau observed in the reward function for dose differences below −1000 mg and above 1000 mg suggests that, in these ranges, deviations from the model’s dose recommendations were not associated with corresponding deviations from achieving the trough goal. These cases may reflect clinical complexities not captured by the model, warranting further evaluation. Despite including variables to capture trends in vital and laboratory measurements, our model exhibited high prediction error in patients experiencing acute changes in kidney function. The inclusion of a comprehensive set of AKI risk factors and predictors of low SCr could help reduce the prediction error in these patients [48, 49]. We have previously developed a model to predict next-day SCr in critically ill patients [50]. Incorporating anticipated SCr trends could further enhance predictive accuracy in these cases. Finally, although the limited number of measurements available precluded the development of an AUC-based model, Nephrocast-V was trained on steady-state trough estimates derived from a Bayesian pharmacokinetic framework. Future efforts will focus on addressing these limitations and improving clinical utility by incorporating the capability of predicting AUC within 24 h.

## 5 | Conclusion

In this study, we developed and validated an RNN model to predict vancomycin trough levels and recommend personalized dosing adjustments for ICU patients. The model demonstrated consistent performance by effectively integrating diverse clinical features, including patient-specific variables, and achieving low prediction errors comparable to those of a clinical pharmacist. The model’s ability to recommend vancomycin dosing adjustments optimized for therapeutic trough concentration underscores its potential to serve as a clinical decision support tool for clinical pharmacists.

## Supplementary Material

sup

Additional supporting information can be found online in the Supporting Information section. Appendix S1: phar70062-sup-0001-AppendixS1.docx.

## Figures and Tables

**TABLE 1 |:** Patient characteristics.

Variable	Training (*N* = 1761)	Test (*N* = 444)

Vancomycin levels, (*n*)	1986	506
Age, median (IQR), years	57.86 (43.4–67.57)	57.74 (42.27–66.71)
Sex, *n* (%)		
Male	1115 (63.32)	290 (65.32)
Female	646 (36.68)	154 (34.68)
Ethnicity, *n* (%)		
Black	127 (7.21)	27 (6.08)
White	919 (52.19)	223 (50.23)
Asian	112 (6.36)	29 (6.53)
Other	603 (34.24)	165 (37.16)
SOFA Score, median (IQR)	6 (4–9)	6 (4–9)
ICU length of stay, median (IQR), days	4.9 (2.8–7.9)	4.9 (2.8–7.8)
Unit type, *n* (%)^a^		
MICU	498 (28.3)	149 (33.6)
SICU	969 (55.0)	218 (49.1)
Other	294 (16.7)	77 (17.3)
ICU mortality, *n* (%)	156 (8.9)	37 (8.3)
Baseline SCr (mg/dL), median (IQR)^b^	0.63 (0.48–0.81)	0.63 (0.48–0.83)
AKI, stage, *n* (%)^c^		
I	511 (29.0)	121 (27.3)
II	209 (11.9)	60 (13.5)
III	42 (2.4)	13 (2.9)
Comorbidities, *n* (%)		
Anemia	119 (6.8)	30 (6.8)
Chronic kidney disease	54 (3.1)	12 (2.7)
Coronary artery disease	119 (6.8)	30 (6.8)
Diabetes	192 (10.9)	50 (11.3)
Hypertension	261 (14.8)	67 (15.1)
Liver disease	3 (0.2)	3 (0.7)
Severe sepsis/Septic shock	287 (16.3)	81 (18.2)

Abbreviations: AKI, acute kidney injury; ICU, intensive care unit; MICU, medical intensive care unit; SCr, serum creatinine; SICU, surgical intensive care unit; SOFA, sequential organ failure assessment.

a Patients may be transferred between different units during their hospitalization.

b Baseline serum creatinine (SCr) was defined as the first measurement obtained during the hospital stay.

c Acute kidney injury (AKI) was identified based on the 2012 Kidney Disease Improving Global Outcomes (KDIGO) criteria, utilizing the ratio of peak to baseline SCr [29].

**TABLE 2 |:** Summary of model performance.

	Train dataset		
	Nephrocast-V	Pharmacist dosing	*p*

MAE (mg/L)	3.15	3.21	0.592
RMSE (mg/L)	4.16	4.44	0.051
MAPE	20.96	22.54	0.140

	Test dataset		
	Nephrocast-V	Pharmacist dosing	*p*

MAE (mg/L)	3.15	3.06	0.732
RMSE (mg/L)	4.17	4.07	0.751
MAPE	20.45	18.6	0.325

Abbreviations: MAE, mean absolute error; MAPE, mean absolute percentage error; RMSE, root mean squared error.

**TABLE 3 |:** Assessment of model accuracy in the test dataset.

	Measured trough		
	Subtherapeutic	Therapeutic	Supratherapeutic

*Nephrocast-V*			
Subtherapeutic	10	14	4
Therapeutic	6	33	9
Supratherapeutic	1	2	2
*Pharmacist dosing*			
Subtherapeutic	11	13	3
Therapeutic	6	35	12
Supratherapeutic	0	1	0

*Note:* Confusion matrices comparing predicted trough therapeutic categories from Nephrocast-V and pharmacist dosing versus measured trough category. Each cell indicates the number of cases falling into the corresponding predicted versus measured trough category.

**TABLE 4 |:** Error analysis conducted by a pharmacist.

Patient	Potential cause of error

1	AKI resolved after starting vancomycin
2	AKI resolved after starting vancomycin
3	Patient has paraplegia
3	Patient has quadriplegia
5	Patient has super morbid obesity
6	Low serum creatinine at baseline for unknown reason
7	Patient has paraplegia and diagnosed with malnutrition
8	Recipient of kidney transplant
9	Patient was on end-of-life care
10	Patient developed AKI after starting vancomycin

## Data Availability

The data that support the findings of this study are available on request from the corresponding author. The data are not publicly available due to privacy or ethical restrictions. No public repository exists for the study data, as it contains protected health information.

## Appendix A

### Assessment of Model Performance

Mean absolute error (MAE), root mean squared error (RMSE), and mean absolute percentage error (MAPE) were calculated using the following equations:

$$\text{MAE}=\frac{\sum_{i=1}^{N}|{\;\text{Predicted Vancomycin Trough}}_{i}-{\;\text{Measured Vancomycin Trough}}_{i}|}{N}\;\text{RMSE}=\sqrt{{\left.\frac{\sum_{i=1}^{N}\left({\text{Predicted Vancomycin Trough}}_{i}-\right.\;Measured\;Vancomycin\;Trough_{i}\;}{N}\right)}^{2}}\;\text{MAPE}\;=\frac{1}{N}\sum_{i=1}^{N}\left|\frac{{\;\text{Predicted Vancomycin Trough}}_{i}-{\;\text{Measured Vancomycin Trough}}_{i}}{{\;\text{Measured Vancomycin Trough}}_{i}}\right|$$

Here, N is the number of measurements assessed.

## Appendix B

### Dosage Recommendations

The dose recommendation reward function is defined as follows.

$$R(t)=\frac{k}{1+e^{-(t-l)}}-\frac{k}{1+e^{-(t-u)}}$$

Here, t is the vancomycin trough concentration, and l and u are the lower and upper bounds of the trough goal range. The constant k is used to scale the reward function within the range [0, 1]. This function assigns values close to 1 to the trough values that are within the goal range and values closer to 0 to those outside the therapeutic range. The reward decreases exponentially for sub-therapeutic levels (below the lower bound) as the trough deviates further from the target range. Similarly, for supratherapeutic levels (above the upper bound), the reward also decreases exponentially as the deviation increases. This ensures that the reward is highest for values within the therapeutic range and penalizes both excessively low and excessively high trough levels.
