# Supplementary material for: Nephrocast-V: A Deep Learning Model for the Prediction of Vancomycin Trough Concentration Using Electronic Health Record Data
Source: Pharmacotherapy. Author manuscript; Available in PMC 2025 Dec 5. (PMC12674970; doi:10.1002/phar.70062)

**Supplementary Information**

**Table S1**: List of model features.

| **Variable** | **Unit** | **Variable** | | **Unit** |
| --- | --- | --- | --- | --- |
| Vital Signs (11) | | | | |
| Heart rate | beats/minute | Diastolic blood pressure | | mmHg |
| Pulse Oximetry | % | Mean arterial pressure | | mmHg |
| Temperature | degrees Celsius | Respiration rate | | breaths/minute |
| Systolic blood pressure | mmHg | End tidal CO2 | | mmHg |
| Weight | kg | Height | | inches |
| Body mass index | kg/m² |  | |  |
| Laboratory Values (42) | | | | |
| Excess bicarbonate | mmol/L | Hemoglobin | | g/dL |
| Bicarbonate | mmol/L | Partial Thromboplastin Time | | seconds |
| Fraction of inspired Oxygen | % | White Blood Cell count | | count*10^3 /µL |
| pH | - | Fibrinogen | | mg/dL |
| Partial pressure of CO2 from arterial blood | mmHg | Platelets | | count*10^3 /µL |
| Oxygen saturation from arterial blood | % | Partial pressure of O2 from arterial blood | | mmHg |
| Aspartate transaminase | IU/L | Brain natriuretic peptide | | ng/L |
| Blood Urea Nitrogen | mg/dL | Sodium | | mg/dL |
| Alkaline phosphate | IU/L | Procalcitonin | | ng/mL |
| Calcium | mg/dL | C-reactive protein | | mg/L |
| Chloride | mmol/L | Ammonia | | mg/L |
| Creatinine | mg/dL | Lymphocytes | | count*10^3 /µL |
| Bilirubin direct | mg/dL | Albumin | | g/dL |
| Serum Glucose | mg/dL | Alanine aminotransferase | | IU/L |
| Lactic acid | mg/dL | Ferritin | | ng/mL |
| Magnesium | mmol/dL | Lactate dehydrogenase | | IU/L |
| Phosphate | mg/dL | Lymphocytes differential | | % |
| Potassium | mmol/L | D dimer | | mcg/mL |
| Total Bilirubin | mg/dL | Red cell width | | um |
| Troponin I | ng/mL | Sedimentation rate | | mm/hr |
| Hematocrit | % | Hemoglobin A1C | | % |
| Demographics (6) | | | | |
| Age | Years | Hours between hospital admit and ICU admit | | hours |
| Gender | Male/Female | Duration until current time | | hours |
| Care Units | Medical/Surgical ICU unit | Mechanical ventilation | | yes/no |
| SIRS/SOFA criteria (11) | | | | |
| Temperature SIRS criteria | yes/no | Liver SOFA score | | [0-4] |
| Respiratory SIRS criteria | yes/no | Cardiovascular SOFA score | | [0-4] |
| Heart rate SIRS criteria | yes/no | Perfusion SOFA score | | [0-4] |
| White blood count SIRS criteria | yes/no | Renal SOFA score | | [0-4] |
| Respiratory SOFA score | [0-4] | CMS SOFA score | | [0-4] |
| Coagulation SOFA score | [0-4] | -- | | -- |
| Medications (12) | | | | |
| Category | Names | Category | | Names |
| Anesthesia | propofol, thiopental sodium, desflurane, sevoflurane, propoven, pentothal, midazolam, methoxyflurane, isoflurane, ketamine, halothane, amidate, etomidate, brevital, methohexital, enflurane, diprivan, ethernhalant, ethrane, enflurane, fospropofol, dexmedetomidine, lorazepam | Antihypertensive | | Esmolol, labetalol, clevidipine, nicardipine |
| Anticoagulants | apixaban, dabigatran, dalteparin, edoxaban, enoxaparin, fondaparinux, heparin, rivaroxaban, warfarin, bivalirudin, argatroban | Antiarrhythmic | | Amiodarone, lidocaine, diltiazem, procainamide |
| Anticonvulsants | topiramate, zonisamide , brivaracetam, carbamazepine, clobazam, clonazepam, divalproex, eslicarbazepine, magnesium sulfate, midazolam, ethosuximide, ethotoin, ezogabine, felbamate, fosphenytoin, gabapentin, lacosamide, lamotrigine, levetiracetam, mephenytoin, mephobarbital, methsuximide, oxcarbazepine, paramethadione, perampanel, phenacemide, phenytoin , pregabalin, primidone, rufinamide, tiagabine, topiramate, trimethadione, valproate sodium, valproic acid, vigabatrin, zonisamide, diazepam, phenobarbital, lorazepam | Prostacyclin | | Epoprostenol |
| Antipsychotics | Haloperidol, olanzapine, quetiapine, risperidone, trifluoperazine | Neuro block | | Cisatracurium, rocuronium, vecuronium |
| Bleeding reversal agents | Kcentra, vitamin k, tranexamic acid, aminocaproic acid, feiba,  Novoseven, xyntha, mononine, humate, advate, profilnine, benefix | Pain meds | | Lidocaine, ketamine, hydromorphone, morphine, fentanyl |
| Vasodilators | Nitroglycerin, nitroprusside | Vancomycin | | Dose  Concentration  Time since last dose |
| Comorbidities (62) | | | | |
| Intravascular coagulation | | | Chronic kidney disease | |
| Necrotizing fasciitis | | | Plasma protein disorder | |
| Tumor lysis syndrome | | | Aplasia | |
| Shock | | | Aspiration pneumonitis | |
| Metastatic cancer | | | Acute myocardial infarction | |
| ST-elevation myocardial infarction | | | Congestive heart failure | |
| Myeloblastic leukemia | | | Encephalopathy | |
| Liver failure | | | Thrombocytopenia | |
| Malignant pleural effusion | | | Coronary artery disease | |
| Pneumothorax | | | Chronic obstructive pulmonary disease | |
| Cachexia | | | Diabetes | |
| Coma | | | Human immunodeficiency virus | |
| Peritonitis | | | Hypertension | |
| Lymphoma | | | Obesity | |
| Carditis | | | Severe brain condition | |
| Septic embolism | | | Other pulmonary condition | |
| Coagulation defect | | | Non-rheumatic valve disease | |
| Primary lung cancer | | | Urinary tract infection | |
| Lymphoid leukemia | | | Anemia | |
| Pulmonary embolism | | | Pneumonia | |
| Pericardial effusion | | | Hyperlipidemia | |
| Cerebral infarction | | | Major depressive disorder | |
| Meningitis | | | Bacteremia | |
| Malignant liver cancer | | | Gastro-esophageal reflux disease | |
| Gastrointestinal hemorrhage | | | Hypothyroidism | |
| Acute renal failure | | | Long term use of insulin | |
| Chronic liver disease | | | Liver cirrhosis | |
| Neutropenia | | | Immune conditions | |
| Cardiac arrhythmia | | | Solid malignancy | |
| Malnutrition | | | Organ transplant | |
| Pulmonary heart disease | | | Rheumatic condition | |

ICU = Intensive care unit; SIRS = Systemic Inflammatory Response Syndrome; SOFA = Sequential Organ Failure Assessment.

**Figure S1**: Bland–Altman plots of measured and predicted vancomycin trough concentrations by a pharmacists aided by Bayesian dosing software in the test dataset.


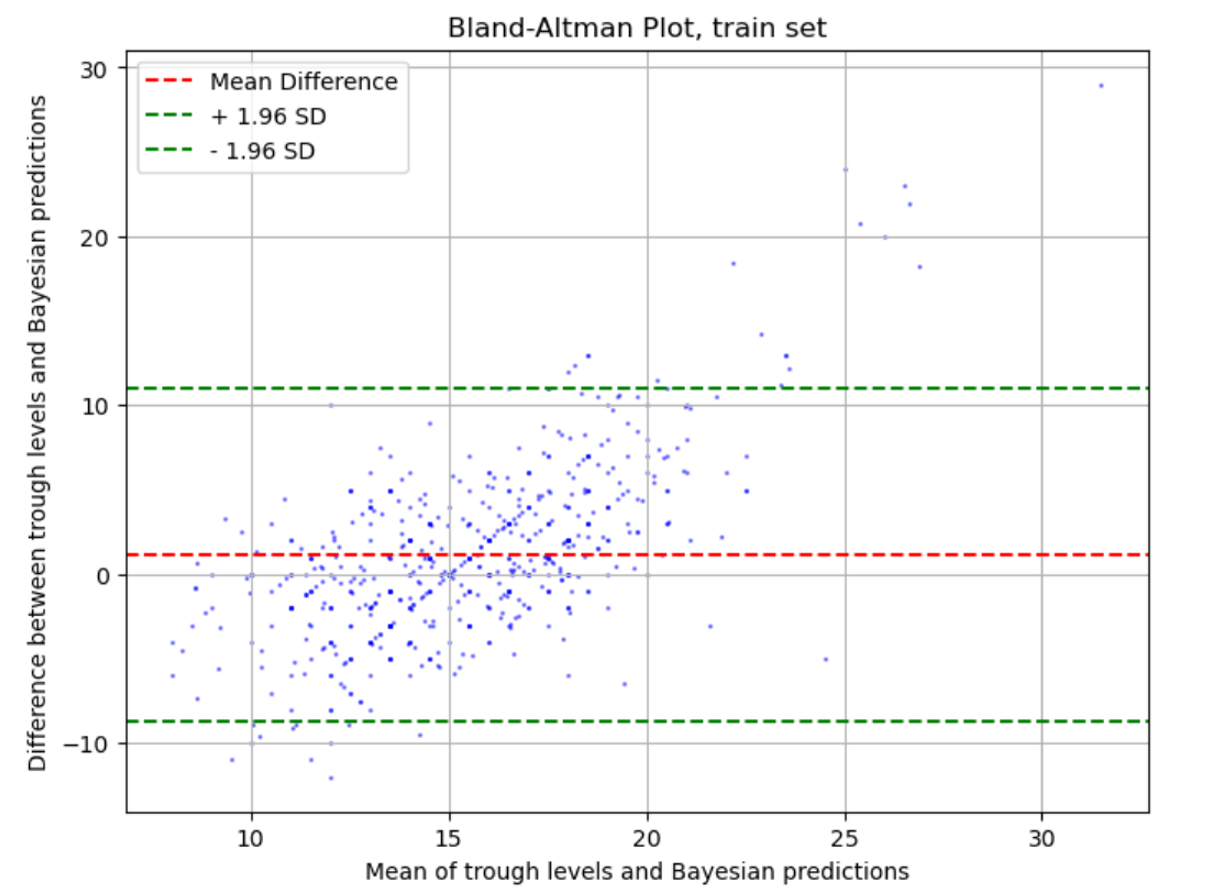

Supplement: sup [file NIHMS2116190-supplement-sup.docx]
